# Visitation patterns of two ray mesopredators at shellfish aquaculture leases in the Indian River Lagoon, Florida

**DOI:** 10.1371/journal.pone.0285390

**Published:** 2023-05-04

**Authors:** Brianna V. Cahill, Breanna C. DeGroot, Lauran R. Brewster, Steven M. Lombardo, Charles W. Bangley, Matthew B. Ogburn, Matthew J. Ajemian

**Affiliations:** 1 Harbor Branch Oceanographic Institute, Florida Atlantic University, Fort Pierce, Florida, United States of America; 2 School for Marine Science and Technology, University of Massachusetts Dartmouth, New Bedford, Massachusetts, United States of America; 3 Bonefish and Tarpon Trust, Miami, Florida, United States of America; 4 Smithsonian Environmental Research Center, Edgewater, Maryland, United States of America; 5 Dalhousie University, Halifax, Nova Scotia, Canada; MARE – Marine and Environmental Sciences Centre, PORTUGAL

## Abstract

The Indian River Lagoon is a primary location of field-based “grow-out” for bivalve shellfish aquaculture along Florida’s Atlantic coast. Grow-out locations have substantially higher clam densities than surrounding ambient sediment, potentially attracting mollusk predators to the area. Inspired by clammer reports of damaged grow-out gear, we used passive acoustic telemetry to examine the potential interactions between two highly mobile invertivores—whitespotted eagle rays (*Aetobatus narinari*) and cownose rays (*Rhinoptera* spp.)—and two clam lease sites in Sebastian, FL and compared these to nearby reference sites (Saint Sebastian River mouth, Sebastian Inlet) from 01 June 2017 to 31 May 2019. Clam lease detections accounted for 11.3% and 5.6% of total detections within the study period, for cownose and whitespotted eagle rays, respectively. Overall, the inlet sites logged the highest proportion of detections for whitespotted eagle rays (85.6%), while cownose rays (11.1%) did not use the inlet region extensively. However, both species had significantly more detections at the inlet receivers during the day, and on the lagoon receivers during the night. Both species exhibited long duration visits (> 17.1 min) to clam lease sites, with the longest visit being 387.5 min. These visit durations did not vary substantially between species, although there was individual variability. Based on generalized additive mixed models, longer visits were observed around 1000 and 1800 h for cownose and whitespotted eagle rays, respectively. Since 84% of all visits were from whitespotted eagle rays and these longer visits were significantly longer at night, this information suggests that observed interactions with the clam leases are potentially underestimated, given most clamming operations occur during daytime (i.e., morning). These results justify the need for continued monitoring of mobile invertivores in the region, including additional experimentation to assess behaviors (e.g., foraging) exhibited at the clam lease sites.

## Introduction

Currently, at least 89% of the world’s shellfisheries come from aquaculture operations [1]. While these operations are becoming more common in coastal communities throughout the world [2], large scale shellfish deployments can affect the community structure of marine environments. Shellfish aquaculture operations attract a variety of sessile and mobile organisms due to the contribution of both physical structure, similar to that of artificial reef habitats [3], and trophic subsidies in high-density lease areas [3]. For example, blue mussel (*Mytilus edulis*) suspended aquaculture farms in Canada increased total species abundance within 50 m of the lease edges [4]. Conversely, assessments of on-bottom cultures of the Peruvian bay scallop (*Argopecten purpuratus*) resulted in a negative substantial change in the benthic community composition after scallop cultures were deployed [5]. There, changes in ecosystem function through bottom-up trophic shifts and decreases in overall biodiversity were observed, driven by large increases in gastropod predator abundances [5]. These studies highlight variation in how community structure can shift due to shellfish aquaculture.

While most research to date has focused on benthic or abundant species (e.g., aquaculture interactions, community composition), the interactions of less common or highly mobile marine predators with shellfish aquaculture operations are not well established. Durophagous stingrays (e.g., Rhinopteridae, Myliobatidae, and Aetobatidae) are examples of highly mobile predators with the capacity to interact with molluscan shellfish enhancement activities. Rhinopterid diets have been contested as both opportunistic generalist [6] and habitat-specific [7], consuming crustaceans, polychaetes, echinoderms and mollusks [6, 7]. Conversely, aetobatid diets are largely considered specialist [8, 9], consuming primarily mollusks and crustaceans. However, species within these families have diets that both include hard-shelled mollusks [7, 8, 10–12] and spend a significant portion of their time within coastal estuaries and lagoons, where aquaculture activities are typically situated [11, 13, 14]. Cownose rays (*Rhinoptera bonasus*) in particular have long been considered as potential threats to bivalve shellfish (i.e., eastern oyster (*Crassostrea virginica*) and bay scallop (*Argopecten irradians*)) along the US East Coast [12, 15–17], to the point in which there was growing support for culling efforts by commercial fisherman [15] and the development of a cownose ray fishery [16, 18] in the Chesapeake Bay region. However, numerous studies examining cownose ray diet provided evidence that commercially important bivalves comprise a minimal, if any, proportion of the diet [6, 7, 12, 19]. Nevertheless, population control efforts on K-selected species (e.g., cownose rays [20]) with extremely low reproductive potential has been contested [21], though cownose ray populations have never been formally assessed. Similarly, gear damage has been observed for other bivalve aquaculture operations in French Polynesia, implicating spotted eagle rays (*Aetobatus ocellatus*) [22, 23], but again interactions have been poorly described.

Since visual observations of ray interactions within shellfish enhancement habitats are unpredictable given the dynamic nature of these predators and environmental constraints (e.g., visibility), other techniques such as biotelemetry can be employed to better understand potential interactions. Passive acoustic telemetry, whereby acoustically tagged animals are detected by arrays of moored receivers, has grown tremendously in popularity and is now a widely applied tool to reveal animal behaviors in the aquatic environment [24]. Due to the expansive use of receiver arrays and networks [25] and collaborative opportunities to track animals at large scales, cooperative networks have broadly emerged worldwide, providing insight into seasonal movements and large-scale migrations of multiple species, including durophagous stingrays [26–28]. Fewer studies have utilized this approach to examine visitation patterns by these rays across estuaries or at distinct shellfish enhancement sites. Only a single recent study applied acoustic telemetry to monitor predators at a shellfish enhancement area [29], although this was assessed using more sedentary predators (i.e., channeled (*Busycotypus canaliculatus*) and knobbed whelk (*Busycon carica*)) on a relatively small experimental site.

The Indian River Lagoon (IRL) is a primary location for hard clam (*Mercenaria mercenaria*) aquaculture operations on Florida’s Atlantic coast. Using designated underwater acreage known as clam leases, clams are grown in artificially high-density plots (50–70 clams per square foot [30]), from seed (10–15 mm) [31] to littleneck size (2.5 cm) [30] or larger. The grow-out phase can take from 12 to 24 months [30] depending on clam harvest size. Hard clam aquaculture has two main grow-out strategies including bagged planting, where clams are placed into 1.2 m x 1.2 m polyester mesh clam bags on the sediment surface [32] and bottom planting, where clams are able to bury and a large sheet of protective cover netting (e.g., high density polyethylene (HDPE) or chicken wire) is staked over top [30]. Given the high densities of clams within these confined areas, and the ability of whitespotted eagle rays to manipulate shellfish aquaculture gear [33], both whitespotted eagle rays (*A*. *narinari*) and cownose rays (*Rhinoptera* spp.) are suspected to interact with shellfish aquaculture grow-out sites.

Clam fishermen have reported the presence of rays within lease areas designated for shellfish aquaculture, and have suggested that gear damage, such as torn bags and crushed clams, may be a result of ray interactions (E. Mangano, Orchid Island Shellfish, Inc., pers. comm). Unfortunately, few studies have assessed the occurrence patterns of these predators, which limits our understanding of the extent of these interactions. DeGroot et al. [14] acoustically tracked seven whitespotted eagle rays within the IRL and found that the rays frequented areas of high anthropogenic use, such as boat channels and inlets. These habitats were used more frequently during the day, whereas shallower lagoon habitats were used more at night. Lagoon use varied by individual, but only the single female ray tracked exhibited partial habitat use over the clam leases within the Sebastian region. Limited tracking durations provided by active acoustic telemetry confirmed the use (and re-use) of these clam lease habitats [14]; however, the extent to which whitespotted eagle rays utilize and interact with clam lease habitats could not be determined without long-term observation of multiple individuals. As such, longitudinal monitoring is necessary to investigate and quantify ray interactions with shellfish aquaculture operations.

Though passive acoustic telemetry is primarily used to understand large scale movements, receivers can be placed strategically to understand visitation patterns of tagged animals to habitats of particular interest, such as shellfish aquaculture use zones. Here, we employed passive acoustic telemetry techniques to understand how tagged rays used areas designated for shellfish aquaculture operations and compared them to other reference receivers present within the Sebastian, FL region of the IRL over a two-year study period. Using detections from tagged cownose and whitespotted eagle rays, the specific objectives for this study were to (1) quantify frequency of detections at clam lease receivers, (2) assess diel variation in detection patterns for clam lease vs other nearby habitats, (3) assess variation in detection patterns by sex and location, (4) compare visit duration (min) of tagged rays at clam lease habitats to that of other Sebastian receiver locations, and (5) investigate the effect of abiotic factors (temperature, salinity, dissolved oxygen, tide status, time of day, and moon phase) on visit duration. Generalized additive mixed models (GAMMs) were used to assess species and individual level variation. Given both species’ benthopelagic lifestyle [34] and their propensity to feed on bivalves [7, 8, 10–12], we anticipated similar detection and visit responses by both species despite their differences in migratory patterns along the Atlantic coast [26, 28].

## Methods

### Ethics statement

All animal collection and tagging procedures were approved by Florida Atlantic University’s Institutional Animal Care and Use Committees (Animal Use Protocol #A16-16) and Florida Fish and Wildlife Conservation Commission Special Activity License permit numbers (SAL-16-1785-SRP, SAL-17-1785-SRP, SAL-18-1785A-SRP), as well as the Smithsonian Environmental Research Center’s Institutional Animal Care and Use Committees (Animal Use Protocol SERC2017-0512-1). All efforts were made to minimize animal suffering during tagging procedures.

### Animal collection and tagging

Rays were targeted for acoustic tagging within the IRL, near Sebastian, FL. Animals were captured via boat deployed nets, using either a (a) 200 x 3 m gillnet (two 100 m panels of 15.24 and 20.32 cm stretch mesh), (b) 500 x 4 m nylon seine net, or (c) a 200 x 3 m 35.56 cm stretch mesh nylon tangle net [28]. Once captured, the animal was moved into an onboard live-well containing a free-flowing bilge pump to supply oxygenated ambient seawater. Only animals that were perceived to be in excellent condition (normal coloration and ventilation rates, responsive, etc.) received an InnovaSea Systems, Inc. passive acoustic coded transmitter tag (V13-1H, 30.5 mm x 13 mm, 60–180s transmission rate or V16-4H, 68 mm x 16 mm, 30–90s transmission rate), following injection of a 2% lidocaine hydrochloride solution. Tags were surgically implanted into the right side of the coelomic cavity for both species, as the left ovary is primarily used for reproduction [9]. The surgical procedure involved inverting the ray so that it lay ventrodorsally to induce tonic immobility [35], swabbing with 10% povidone-iodine, making a 2–3 cm incision with a dissection scalpel, inserting the tag, and closing the incision area with two sutures using a surgeon’s knot [14, 28]. Rays were monitored in the onboard live-well for up to three minutes to ensure recovery, prior to release. Though not required Overall, 25 whitespotted eagle rays and 30 cownose rays were tagged and released within the IRL between September 2016 and March 2019.

### Study location and acoustic telemetry

**Fig 1:**
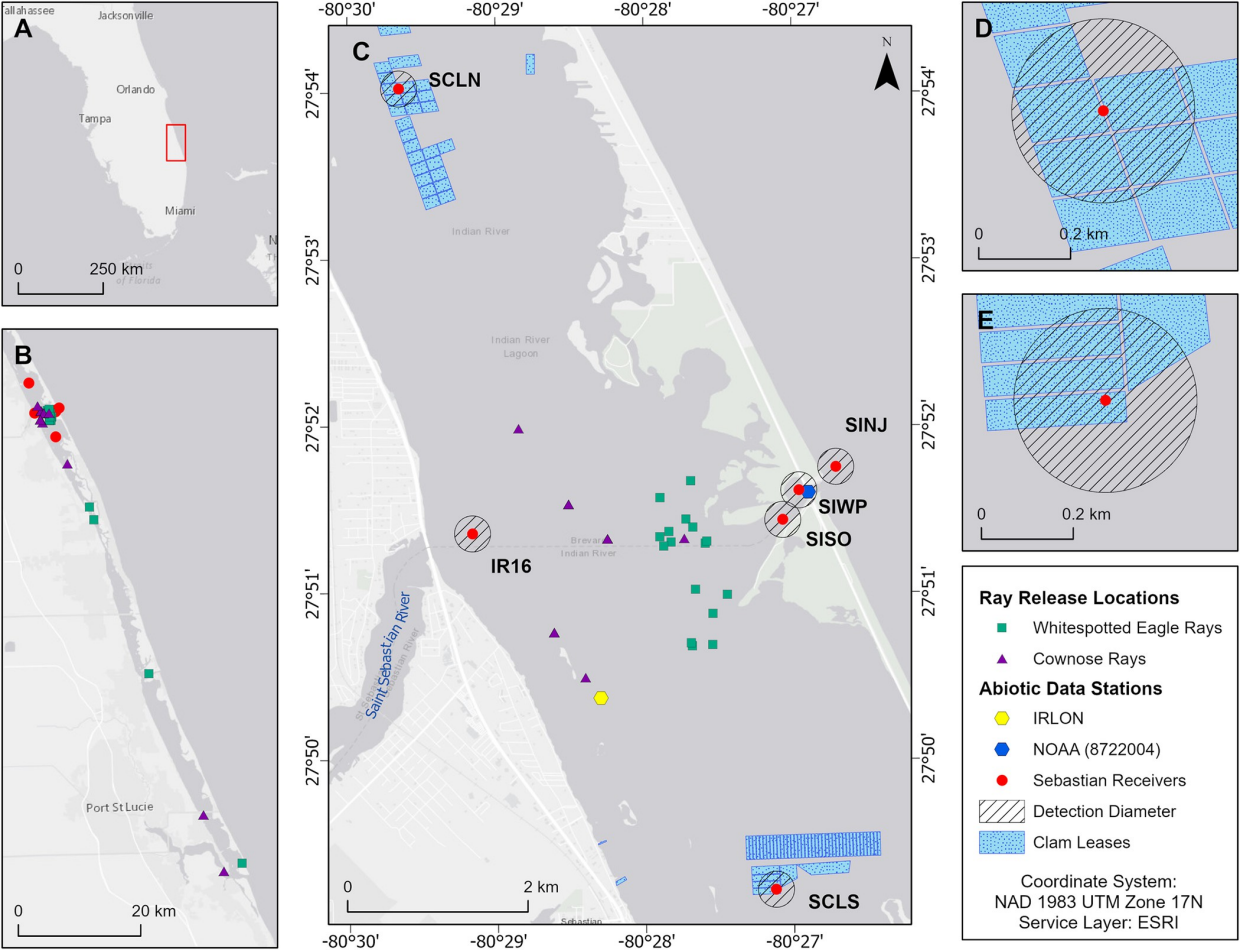
Map showing A) the study region denoted by the red rectangle placed on the Atlantic Coast of Florida, B) capture locations of study individuals between Sebastian, FL and Jensen Beach, FL, C) acoustic receiver locations, including 200 m detection radii for clam lease sites, D) Sebastian clam lease north (SCLN) and E) Sebastian clam lease south (SCLS). Reprinted from Esri “Light Gray Canvas Map” under a CC BY license, with permission from Esri, original copyright.

The Sebastian region of the IRL (Fig 1) is a mosaic of sand flats, mud shallows, mangrove shorelines, and formerly prolific seagrass beds that have decreased over the late 20^th^ century [36]. The region also has a central inlet that allows tidal exchange from the Atlantic Ocean and is influenced by freshwater inputs from the Saint Sebastian River. The variation in benthic composition and hydrology makes the Sebastian area highly dynamic and productive region. Molluscan biodiversity has not been thoroughly characterized in the IRL since the early 1980s, with areas near Sebastian, FL having diverse communities of mollusks (e.g., gastropods, bivalves, nudibranchs) [37]. Of the 428 species observed, Mikkelsen et al. [37] documented hard clams (*Mercenaria mercenaria*) throughout the entirety of the IRL, therefore supporting the suitability for the IRL a primary location for hard clam (*Mercenaria mercenaria*) aquaculture operations along Florida’s Atlantic coast. There are two major Aquaculture Use Zones (AUZs) present within this region that are managed by the Florida Department of Agricultural and Consumer Services. The AUZs, located within the interior of the IRL both north and south of Sebastian Inlet are also surrounded by a variety of individual and in-perpetuity leases. These clam lease locations are generally placed on unstructured bottom (i.e., sand/mud free of seagrass), at 1–2 m in depth, and greater than 30 m distance from both navigational channels [38].

To further understand ray visitation patterns within Sebastian, FL, we coordinated with industry partners (Orchid Island Shellfish Company, Inc.) to deploy two receivers, one within each clam lease habitat—Sebastian Clam Lease North (SCLN) and Sebastian Clam Lease South (SCLS). Orchid Island Shellfish Company, Inc. primarily used bagged planting grow-out methods with an additional layer of HDPE cover netting affixed over top of the bags. An estimated 200 m detection range was selected for all receivers based upon previous research in comparable Florida environments, Sarasota Bay Inlet passes at 134 m [28] and Atlantic inshore habitats at 316 m [39], and balances the potential detection range limitations imposed by variable water depths and benthic habitat complexity. While the detection range of the receivers did not encompass all clam aquaculture sites within each AUZ, receivers were deployed in areas of high farming (i.e., planting and grow-out) activity. These sites were expected to be used throughout the study period and were situated to maximize coverage of active clamming operations without interfering with the operations themselves. As positioned among the clam leases, the receiver detection ranges primarily covered clam lease habitat but also extended out to surrounding habitat (Fig 1). Additionally, acoustic detection data from four other nearby receivers placed in non-clam lease habitats and maintained by other participating FACT Network users (Herndon Solutions Group at Kennedy Space Center and Florida Fish and Wildlife Tequesta), were obtained to compare habitat visitation. One of these receivers was located within the lagoon adjacent to the Saint Sebastian River mouth (IR16), and three were deployed within the Sebastian Inlet spanning East to West (SINJ, SISO and SIWP) (Fig 1). All receivers were near sandy, unstructured bottom, and were active within the study period from June 2017 to June 2019. Receivers (Vemco VR2W or VR2Tx) used to detect tagged animals were semi-permanent fixtures positioned on channel markers, screwed into the seafloor using augers, or were contained within a PVC housing on concrete blocks placed on the seafloor. Acoustic data were downloaded annually using Vemco’s VUE software and raw files were uploaded to the FACT Network data node.

### Data analyses

Acoustic telemetry data were imported into the statistical software R (Version 4.0.4) for analysis. Duplicate and false detections (i.e., false-positive detection data due to tag collision [40]) were filtered manually and removed from the dataset. We determined the proportion of overall use of monitored areas by species, and for each individual, we counted the total number of detections, the total number of days detected, and the number of unique receivers detected upon. To assess diel patterns, detections at all receiver locations were classified as either occurring during the day (0700–1859) or night (1900–0659), with the classifications determined by the average sunrise and sunset times over the study period as given by the *suncalc* package [41]. The spatiotemporal effect on number of detections for each species was modelled using generalized linear mixed models (GLMM) with a Poisson distribution and log link using the package *lme4* [42]. The models were structured with an interaction between time of day, receiver and sex, and a random effect of transmitter ID. For significant categorical effects, detection data were further assessed using a Tukey’s pairwise post-hoc analysis in the package *emmeans* [43].

Visitation events—the amount of consecutive time an individual was detected at a receiver; hereafter referred to as *visits*—were quantified using the *VTrack* package [44]. The minimum criterion for data inclusion using the “RunResidenceExtraction” function requires both a user-defined minimum number of detections and a time-out period. Here, a visit was defined as the total duration that an animal spent within the detection range of a receiver without violating the time-out period. Thus, if a transmitter was detected on a new receiver, or if there was a time difference greater than the time-out period between subsequent detections, the visit was terminated. The minimum criterion for this study was a period in which at least two detections were recorded for an individual tag within the amount of time it would take a ray to pass through an average receiver detection diameter of 400 m. Using the average rate of movement for whitespotted eagle rays of 1.4 km hr^-1^ [14], we calculated a 17.1 min time-out period. This 17.1 min threshold was also used to separate short and long duration visits, the latter of which might suggest interactions with the habitat.

Environmental drivers of visit duration were assessed using publicly available *in situ* and modeled environmental data. Moon fraction data were obtained through the *suncalc* package [41]. Water quality parameters were collected from the Indian River Lagoon Observatory Network of Environmental Sensors (IRLON, irlon.org) Sebastian station for the duration of the study period (Fig 1). Decimal hour of the day was calculated from the datetime information associated with the start time of the visits. Tide status was derived from NOAA’s Tide Predictions using the Sebastian Inlet station (8722004) for the study duration and a tidal lag period—time difference of tidal state between two locations—of two h and 20 min (ΔT) between the Sebastian Inlet and Sebastian downtown region (saltwatertides.com). Since locations for these two regions were not specified on the Saltwater Tide website, SINJ (Sebastian Inlet, East) and IR16 (Sebastian River Mouth) were used as proxies to calculate the distance (3,698 m, *d*) and tidal rate of movement (TRM).

TRM=d/ΔT

Thus, a TRM of 0.439 m/s was calculated. The TRM was then used to determine the tide offset for the two clam lease receivers and the other two inlet receivers. Seasons were determined from each year’s vernal and autumnal equinoxes, and the summer and winter solstices.

A generalized additive mixed effects model (GAMM) using a Gamma distribution and inverse link was built for each species to assess how visit duration was influenced by abiotic drivers: decimal hour, illuminated moon fraction, water temperature, salinity, dissolved oxygen, general location (clam lease vs. other locations), and tidal state. Ray ID was included as a random effect and models were built using the *mgcv* package [45]. GAMMs were selected as they can fit nonlinear relationships between the response variable and covariates [46]. Prior to model building, covariates were assessed for collinearity using correlation plots and variance inflation factors [47]. Model selection was performed using second-order corrected Akaike Information Criterion (AICc) derived from the “dredge” function within the *MuMIn* package [48]. Diagnostic plots provided model validation. The threshold for significance was defined as α = 0.05.

## Results

Of the 55 tagged rays (30 cownose and 25 whitespotted eagle), 38 individuals were detected on at least one of the six receivers within our study area (Table 1). All detected animals were tagged within the IRL spanning from Sebastian, FL to Jensen Beach, FL (Fig 1). Disc width (cm) of the detected animals ranged from 65.0–196.0 cm for whitespotted eagle rays and 46.6–104.0 cm for cownose rays. Female whitespotted eagle rays (n = 8) had a mean disc width of 181.3 ± 5.5 cm, and 143.9 ± 4.2 cm for males (n = 12), whereas female cownose rays (n = 10) had a mean disc width of 91.8 ± 1.2 cm and males (n = 8) at 87.1 ± 2.9 cm. Of the 18 cownose rays detected, eight samples were run for genetic analysis. Four cownose rays were identified as Atlantic cownose rays (*R*. *bonasus*) and four were unexpectedly identified as Brazilian cownose rays (*R*. *brasiliensis*). The remaining cownose rays were assumed to be Atlantic cownose rays. Separate Atlantic and Brazilian cownose ray models were tested; however, insufficient sample sizes prevented GAMM convergence, thus all cownose ray (*Rhinoptera* spp.) visits were pooled for analysis.

**Table 1:** All tagged animals detected within the Sebastian, FL region. Animal numbers corresponding to whitespotted eagle rays and cownose rays have the codes SER and CNR, respectively. All individuals with the superscript “a” (^a^) describe species genetic confirmation. Both species of cownose rays (*R*. *bonasus* and *R*. *brasiliensis*) were merged for visit analysis. Transmitter ID 14583 has a superscript “b” (^b^) as it was the only tagged potential pregnant female detected within the array. Animals caught in the same set (release date) are denoted with the following symbols (*, ^, ^#^). Number of visits refers to the number of unique visits that occurred, not the total time spent.

Species	Animal Number	Tag ID	Release Date	Date of Last Detection	Tagging Location	Sex	DW (cm)	Weight (kg)	Unique Receivers	Days Detected	Number of Detections	Number of Visits
*A*. *narinari*	SER 1	16921	2016-09-20	2018-06-10	Sebastian, FL	F	184.0	96.4	6	118	10,022	543
	SER 2	16922	2016-09-20	2019-05-30	Sebastian, FL	M	153.0	53.6	5	453	30,551	2,541
	SER 3	16923	2016-09-21	2018-11-10	Jensen Beach, FL	F	118.6	23.0	1	1	17	1
	SER 4	16924	2016-09-21	2018-10-31	Fort Pierce, FL	M	124.0	33.8	1	1	50	3
	SER 5	46180	2017-03-10	2017-12-29	Sebastian, FL	M	65.0	2.0	6	39	2,008	89
	SER 6	14587	2017-06-26	2017-07-09	Sebastian, FL	M	134.2	NA	3	7	875	26
	SER 7	14589	2017-07-07	2018-03-29	Vero Beach, FL	F	83.0	NA	6	12	298	24
	SER 8	14584	2017-07-25	2018-10-21	Sebastian, FL	M	151.0	52.8	6	286	22,867	1,402
	SER 9	14585	2017-07-25	2019-04-12	Sebastian, FL	M	127.2	34.4	6	283	17,468	1,115
	SER 10	14586	2017-07-25	2019-04-26	Sebastian, FL	F	156.4	58.4	6	225	13422	872
	SER 11	13488	2017-07-26	2019-05-07	Sebastian, FL	F	195.0	109.4	6	380	25216	1,611
	SER 12	13490	2017-07-26	2018-10-23	Sebastian, FL	F	182.0	11.4	6	279	17924	1,045
	SER 13	84	2017-07-26	2019-04-08	Sebastian, FL	F	186.0	109.1	6	215	6831	731
	SER 14	85	2017-07-26	2019-04-27	Sebastian, FL	M	151.8	52.2	6	458	14661	1,718
	SER 15	86	2017-07-27	2019-05-15	Sebastian, FL	M	128.4	33.6	6	371	9342	1,033
	SER 16	87	2018-04-05	2019-05-29	Sebastian, FL	M	133.8	NA	5	45	3,000	212
	SER 17	7684	2018-06-26	2019-03-21	Sebastian, FL	M	147.8	43.7	6	194	8768	929
	SER 18	7683	2018-07-25	2019-05-31	Sebastian, FL	F	145.2	38.8	6	62	1,146	129
	SER 19	7682	2018-08-13	2018-12-13	Sebastian, FL	M	119.0	22.7	6	57	1547	175
	SER 20	7681	2018-08-21	2019-03-22	Sebastian, FL	M	146.0	48.0	5	36	1,003	118
*R*. *bonasus*	CNR 1	16927	2017-01-10*	2019-03-31	Sebastian, FL	F	71.5	6.0	6	51	1126	93
	CNR 2	16929	2017-01-10*	2019-03-13	Sebastian, FL	F	89.0	12.7	6	109	3246	275
	CNR 3	16930	2017-01-10*	2018-12-12	Sebastian, FL	M	83.0	10.0	6	103	2537	185
	CNR 4	16942	2017-01-11^	2018-01-02	Sebastian, FL	M	89.0	8.89	6	46	559	77
	CNR 5	16943	2017-01-11^	2018-06-07	Sebastian, FL	M	104.0	14.6	6	48	698	93
	CNR 6	16944	2017-01-11^	2018-06-12	Sebastian, FL	F	85.7	10.0	6	95	1413	192
	CNR 7	16945	2017-01-11^	2019-05-21	Sebastian, FL	F	87.9	10.8	6	106	1684	212
	CNR 8	16946	2017-01-11^	2017-06-22	Sebastian, FL	F	95.1	14.0	4	10	131	30
	CNR 9	16947	2017-01-11^	2019-03-04	Sebastian, FL	F	90.2	11.8	6	122	1911	227
	CNR 10^a^	1706	2018-01-11	2018-01-31	Sebastian, FL	M	47.2	1.2	3	4	22	4
	CNR 11^a^	1707	2018-01-12^#^	2018-01-12	Sebastian, FL	M	51.9	1.6	2	1	2	0
	CNR 12^a^	1708	2018-01-12^#^	2018-01-12	Sebastian, FL	F	46.6	1.4	2	1	7	1
	CNR 13^a^	17626	2017-04-19	2018-01-01	Jensen Beach, FL	M	76.0	5.4	6	27	128	27
	CNR 14	15429	2018-03-09	2019-03-22	Jensen Beach, FL	F	80.2	9.2	1	3	13	3
*R*. *brasiliensis*	CNR 15^a^	15428	2018-06-13	2018-12-25	Sebastian, FL	F	95.7	16.2	1	7	30	8
	CNR 16^a^	17624	2017-04-12	2019-01-28	Sebastian, FL	M	87.0	10.5	6	65	344	74
	CNR 17^a, b^	14583	2017-08-07	2019-05-31	Sebastian, FL	F	93.2	13.6	1	248	35,585	1,195
	CNR 18^a^	15423	2018-01-11^#^	2018-02-02	Sebastian, FL	M	61.3	2.7	3	2	6	1

### Detections

The number of detections for a given individual ranged from 2–35,585. Overall, there were a total of 236,458 detections from the six sites over the study duration, of which 79.1% were attributed to whitespotted eagle rays (Table 2). Receiver SINJ had the highest number of detections (122,440), but only 1,795 (1.5%) detections at SINJ were from cownose rays (both *R*. *bonasus* and *R*. *brasiliensis*). Whitespotted eagle rays spent most of their detected time within range of two Sebastian Inlet receivers, SINJ (120,645; 64.5%) and SISO (32,992; 17.6%), followed by the Sebastian River mouth receiver (IR16) with 15,809 detections (8.5%). Atlantic cownose rays spent most of their detected time within range of the northern clam lease (SCLN with 3,607 detections; 26.8%), followed by the Sebastian River receiver (IR16 with 2,779 detections; 20.6). The four remaining receivers accounted for 7,091 detections (47.4%), ranging from 903–2,653 detections. Conversely, 99.0% of Brazilian cownose ray detections were from the IR16 receiver and nearly all came from a single individual (CNR 17; 35,585 IR16 detections).

**Table 2:** Number and proportion of detections at each receiver by species and in total, and number of Ray IDs that were detected at each receiver. There are 18 unique cownose rays that were included in the study; however, one individual (CNR 11) did not exhibit any visits due to insufficient tag detections.

		Brazilian Cownose Ray			Atlantic Cownose Ray			Whitespotted Eagle Ray			
Receiver	Depth (m)	N	Prop. of total	Unique Tags	N	Prop. of total	Unique Tags	N	Prop. of total	Unique Tags	Total Detections
**IR16**	2.0	35,610	99.0%	2	2,779	20.6%	10	15,809	8.5%	16	54,198
**SCLN**	1.5	51	0.1%	1	3,607	26.8%	10	8,197	4.4%	16	11,855
**SCLS**	1.5	115	0.3%	1	1,791	13.3%	9	2,748	1.5%	16	4,654
**SINJ**	4.8	51	0.1%	2	1,744	12.9%	11	120,645	64.5%	20	122,440
**SISO**	2.1	127	0.4%	3	2,653	19.7%	13	32,992	17.6%	18	35,772
**SIWP**	2.1	11	0.1%	2	903	6.7%	13	6,625	3.5%	18	7,539
**Total**		35,965	100.0%	4	13,477	100.0%	14	187,016	100.0%	20	236,458

**Fig 2:**
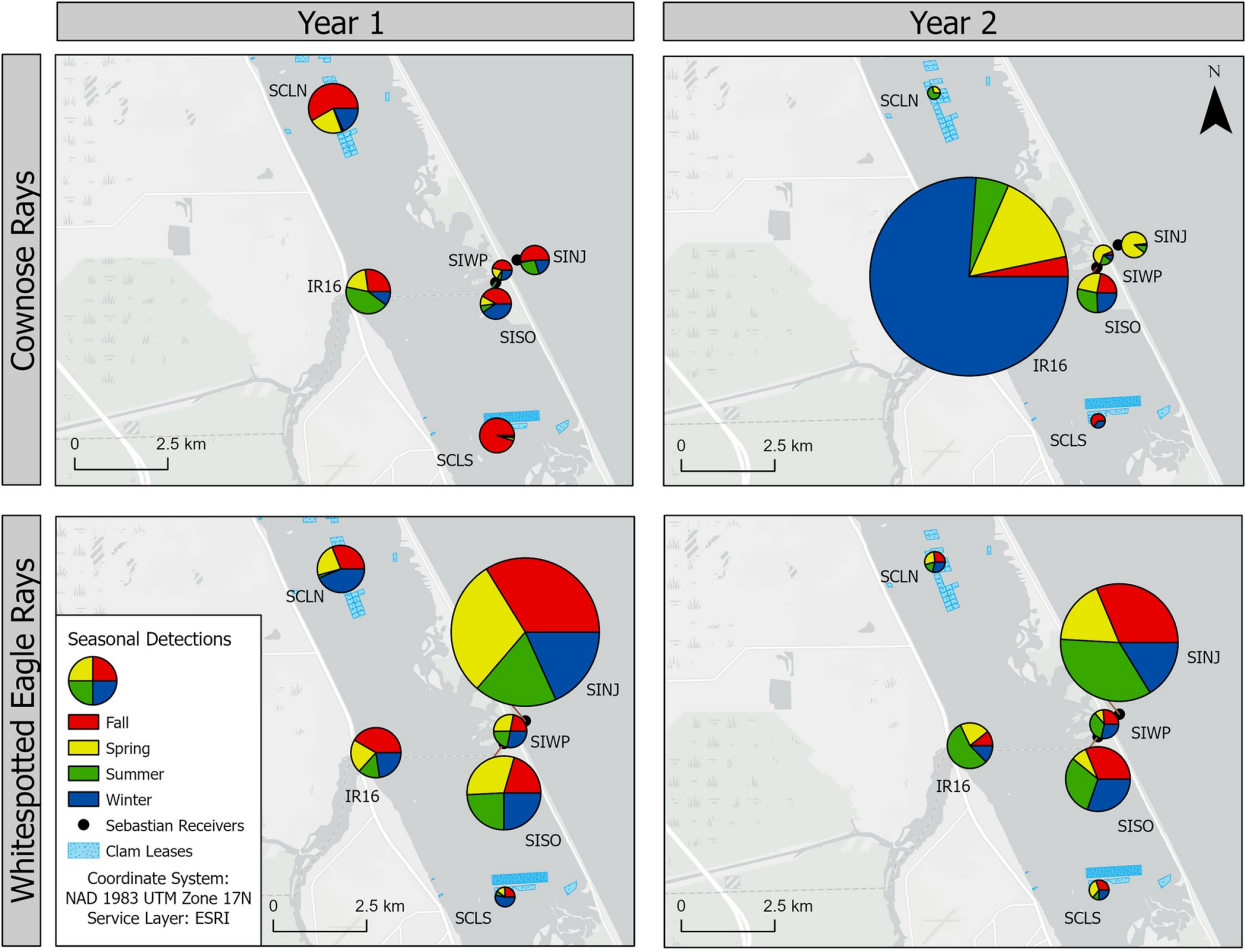
Seasonal detections at each receiver by genus. Year 1 includes June 2017–May 2018 and Year 2 is June 2018–June 2019. Size of pie charts are scaled by the number of detections observed. Reprinted from Esri “Light Gray Canvas Map” under a CC BY license, with permission from Esri, original copyright.

Detections at each receiver varied seasonally and by year. Year 1 spanned 1 June 2017–31 May 2018, and Year 2 spanned 1 June 2018–31 May 2019. For whitespotted eagle rays, the three receivers with the most detections across and within years included SINJ, SISO and IR16 (Fig 2), while SCLS had the fewest detections overall (Table 2). There were minimal differences in proportion of time spent at each receiver between both years; however, there was a decrease in the proportion of detections at SCLN between Year 1 (6.6%) and Year 2 (2.0%). While the inlet (SINJ, SISO, SIWP) and IR16 receivers logged consistent detections in all four seasons, the clam leases logged a substantially smaller proportion of detections within summer months (2.0 and 7.2% of SCLN and SCLS’s yearly detections, respectively) during Year 1. During Year 2, summer detections at the clam leases increased but still accounted for a smaller proportion of total detections than the other seasons (17.1% and 10.8% of SCLN and SCLS’s yearly detections, respectively). IR16 had a large increase in the number of detections between Year 1 and Year 2 (2,822 to 35,567 detections), whereas all other receivers decreased in the number of detections between Year 1 and Year 2 for cownose rays (*Rhinoptera* spp.). Cownose ray inlet detections were inconsistent between years; however, their use of the clam leases was minimal during the summer of Year 1 and decreased overall in Year 2.

Due to unbalanced detection sample sizes among the Brazilian cownose rays, a GLMM could not be fit, thus all station and time of day detection patterns for Brazilian cownose rays could only be interpreted qualitatively. In species-specific GLMMs, sex was not a significant predictor for either Atlantic cownose rays (p = 0.266) or whitespotted eagle rays (p = 0.427); however, both time of day and receiver location were significant predictors for both species (p < 0.001; S1 Table). For both Atlantic cownose and whitespotted eagle rays, the number of detections between day and night were significantly different (p < 0.001); more detections were observed during the day for whitespotted eagle rays (day: 107,777 detections; night: 79,239 detections), whereas there were more detections at night for Atlantic cownose rays (day: 6,657 detections; night: 6,820 detections). Brazilian cownose rays were also detected more frequently at night (day: 13,990 detections; night: 21,975 detections). For Atlantic cownose rays, we observed no significant difference in the overall detection counts between IR16 and SCLN (p = 0.983) or SCLS (p = 0.252), and between SINJ and SISO (p = 0.887). For whitespotted eagle rays, the only combination of receivers that did not have a significant difference in detection counts was observed between SCLN and SISO (p = 0.984). All other Tukey’s post hoc pairwise combinations of receivers were significantly different from one another (p < 0.001).

**Fig 3:**
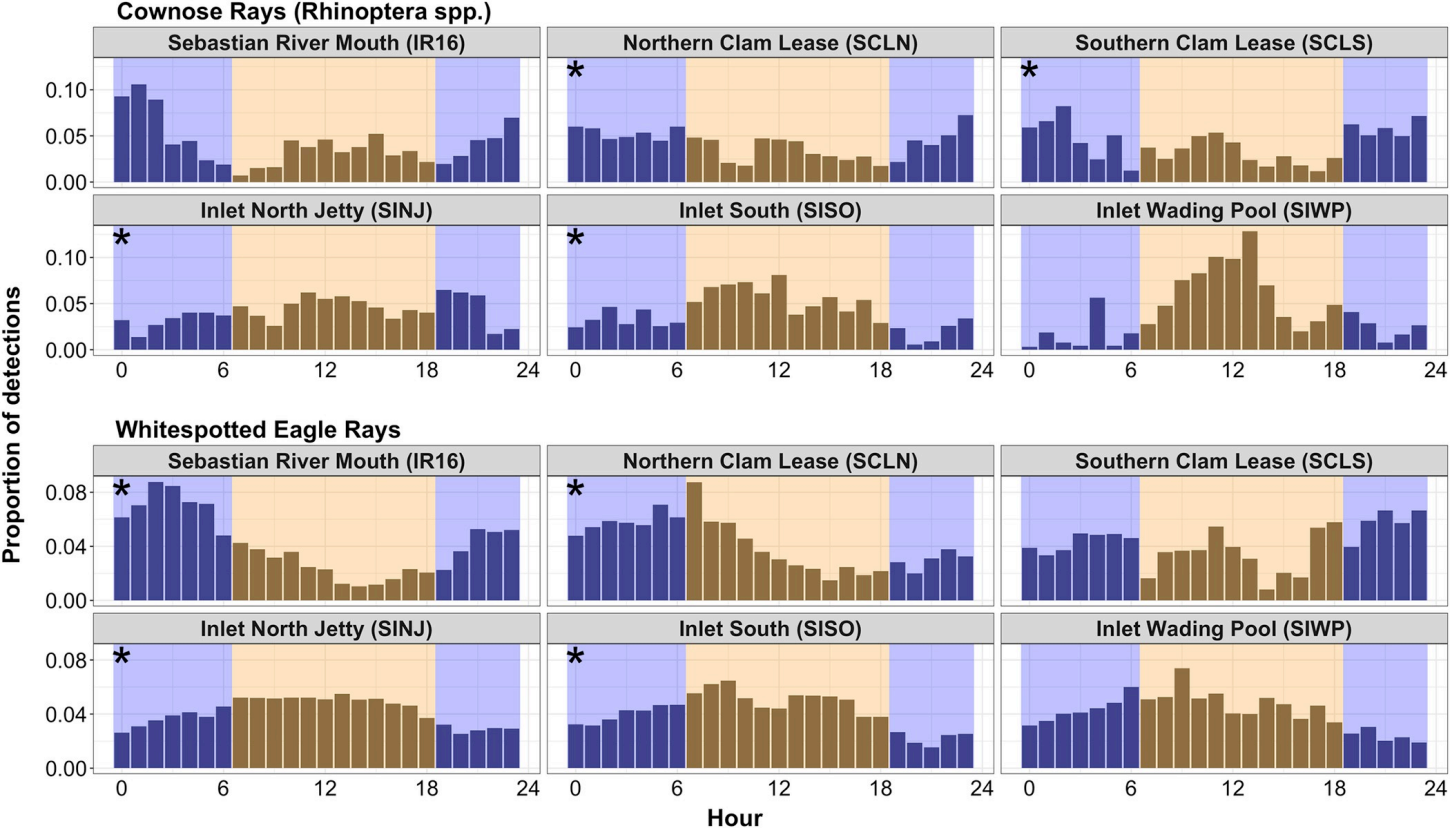
Proportion of hourly detections at each receiver location. Color symbology refers to hours often depicted as night (blue), crepuscular (gray), and day (orange).

Brazilian cownose rays exhibited a greater mean number of detections during the night at IR16 (day: 64.0 ± 0.9 detections; night: 95.8 ± 1.1 detections; Table 3A) and SCLN (day: 4.1 ± 0.7 detections; night: 4.4 ± 0.5 detections), whereas they exhibited a greater mean during the day at all other locations (Table 3A). The greatest overall detection counts were observed during the night at IR16 (day: 13,762 detections; night: 21,848 detections) and SCLS (day: 54 detections; night: 61 detections), whereas the other locations had the greatest detection counts during the day. Atlantic cownose rays exhibited significantly greater mean detection counts during the night at SCLN (day: 12.5 ± 0.4 detections; night: 15.3 ± 0.7 detections; p < 0.001; Table 3B; Fig 3), SCLS (day: 9.8 ± 0.5 detections; night: 13.0 ± 0.5 detections; p < 0.001), and SINJ (day: 10.1 ± 0.6 detections; night: 18.0 ± 0.9 detections; p = 0.002), whereas detections were significantly greater during the day at SISO (day: 8.9 ± 0.3 detections; night: 8.4 ± 0.3 detections). Neither IR16 nor SIWP exhibited significant differences in detection counts during day or night. Whitespotted eagle rays exhibited significantly more detections during the night at IR16 (day: 16.2 ± 0.3 detections; night: 20.1 ± 0.2 detections; p < 0.001; Table 3C) and SCLN (day: 8.0 ± 0.2 detections; night: 9.3 ± 0.2 detections; p < 0.001), and during the day at SINJ (day: 42.9 ± 0.2 detections; night: 32.5 ± 0.2 detections; p < 0.001) and SISO (day: 11.7 ± 0.1 detections; night: 9.5 ± 0.1 detections; p < 0.001). Whitespotted eagle rays did not exhibit a difference in detection counts at SCLS or SIWP. Finally, for both Atlantic cownose and whitespotted eagle rays, there was no significant difference in detection count between males and females by time of day (p > 0.05) or receiver (p > 0.05).

**Table 3:** Mean and total detections (N) at each receiver based on time of day. The tables show the data for A) Brazilian cownose rays, B) Atlantic cownose rays and C) whitespotted eagle rays. Significance was determined by pairwise combinations of day (D) and night (Ni) values.

**A.**	**Day**		**Night**		**Significance**
**Receiver**	**Mean**	**N**	**Mean**	**N**	**D–Ni**
**IR16**	64.0 ± 0.9	13,762	95.8 ± 1.1	21,848	**NA**
**SCLN**	4.1 ± 0.7	29	4.4 ± 0.5	22	**NA**
**SCLS**	10.8 ± 1.2	54	5.1 ± 0.8	61	**NA**
**SINJ**	6.7 ± 1.3	47	4.0 ± 0.0	4	**NA**
**SISO**	3.3 ± 0.3	92	2.5 ± 0.3	35	**NA**
**SIWP**	2.0 ± 0.4	6	1.7 ± 0.5	5	**NA**
**B.**	Day		Night		Significance
**Receiver**	**Mean**	**N**	**Mean**	**N**	**D–Ni**
**IR16**	12.4 ± 0.6	1,031	17.1 ± 0.6	1,748	0.323
**SCLN**	12.5 ± 0.4	1,587	15.3 ± 0.7	2,020	**<0.001**
**SCLS**	9.8 ± 0.5	637	13.0 ± 0.5	1,154	**<0.001**
**SINJ**	10.1 ± 0.6	954	18.0 ± 0.9	790	**0.002**
**SISO**	8.9 ± 0.3	1,784	8.4 ± 0.3	869	**0.016**
**SIWP**	6.9 ± 0.4	664	6.0 ± 0.7	239	1.000
**C.**	Day		Night		Significance
**Receiver**	**Mean**	**N**	**Mean**	**N**	**D–Ni**
**IR16**	16.2 ± 0.3	5,011	20.1 ± 0.2	10,798	**<0.001**
**SCLN**	8.0 ± 0.2	3,969	9.3 ± 0.2	4,228	**<0.001**
**SCLS**	11.0 ± 0.4	1,090	10.7 ± 0.4	1,658	0.291
**SINJ**	42.9 ± 0.2	73,262	32.5 ± 0.2	47,383	**<0.001**
**SISO**	11.7 ± 0.1	20,423	9.5 ± 0.1	12,569	**<0.001**
**SIWP**	4.0 ± 0.1	4,022	3.6 ± 0.1	2,603	0.162

**Fig 4:**
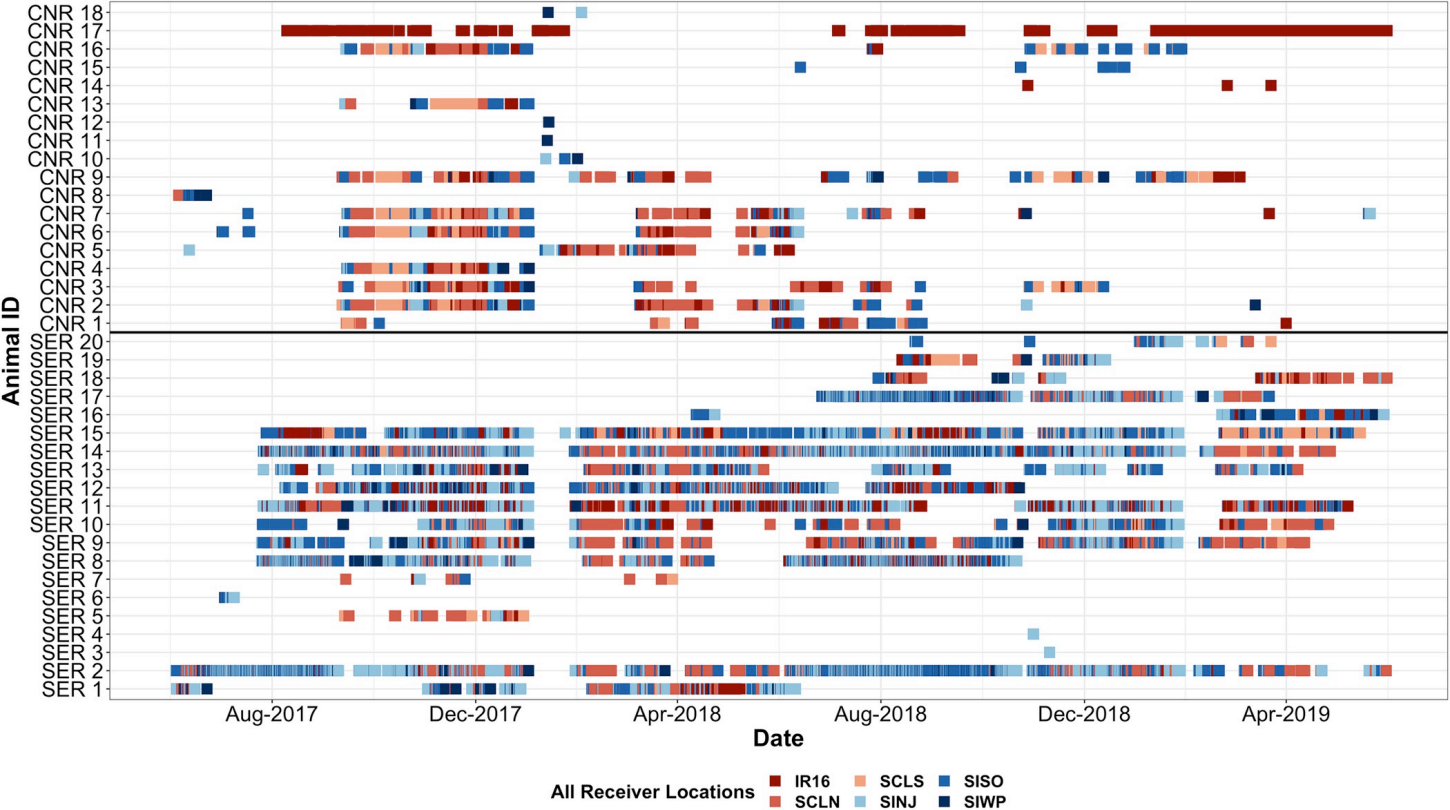
Abacus plot showing detections over time at all receivers. Whitespotted eagle rays (n = 20) and both species of cownose rays (n = 18) exhibit differing movement patterns. Lagoon receivers denoted with red symbology and inlet receivers have blue symbology.

Overall, both species of cownose rays (*Rhinoptera* spp.) were more commonly detected within lagoon habitats whereas whitespotted eagle rays were observed more frequently in the inlet (Fig 4; Table 2). While individual variation was clear, there were periods in which mature cownose rays appeared to move synchronously in between receivers, with instances from September 2017 to January 2018 (CNR 2–4, 6, 7, 9, 13, 16) and again from March 2018 to June 2018 (CNR 2, 3, 5–7, 9), despite being tagged during separate capture events. There were a few individuals (SER 3, 4; CNR 14, 15, 17) that were only detected on a single receiver, though most of those individuals had less than 50 detections (Table 1). During January 2018, whitespotted eagle rays were not detected on any receivers, but seven cownose rays were (CNR 5, 9–12, 17, 18). Overall, there were more unique tag IDs that visited the inlet receivers (33–34) than the lagoon receivers (26–28), which was a consistent pattern for both species (Table 2).

### Visits

**Fig 5:**
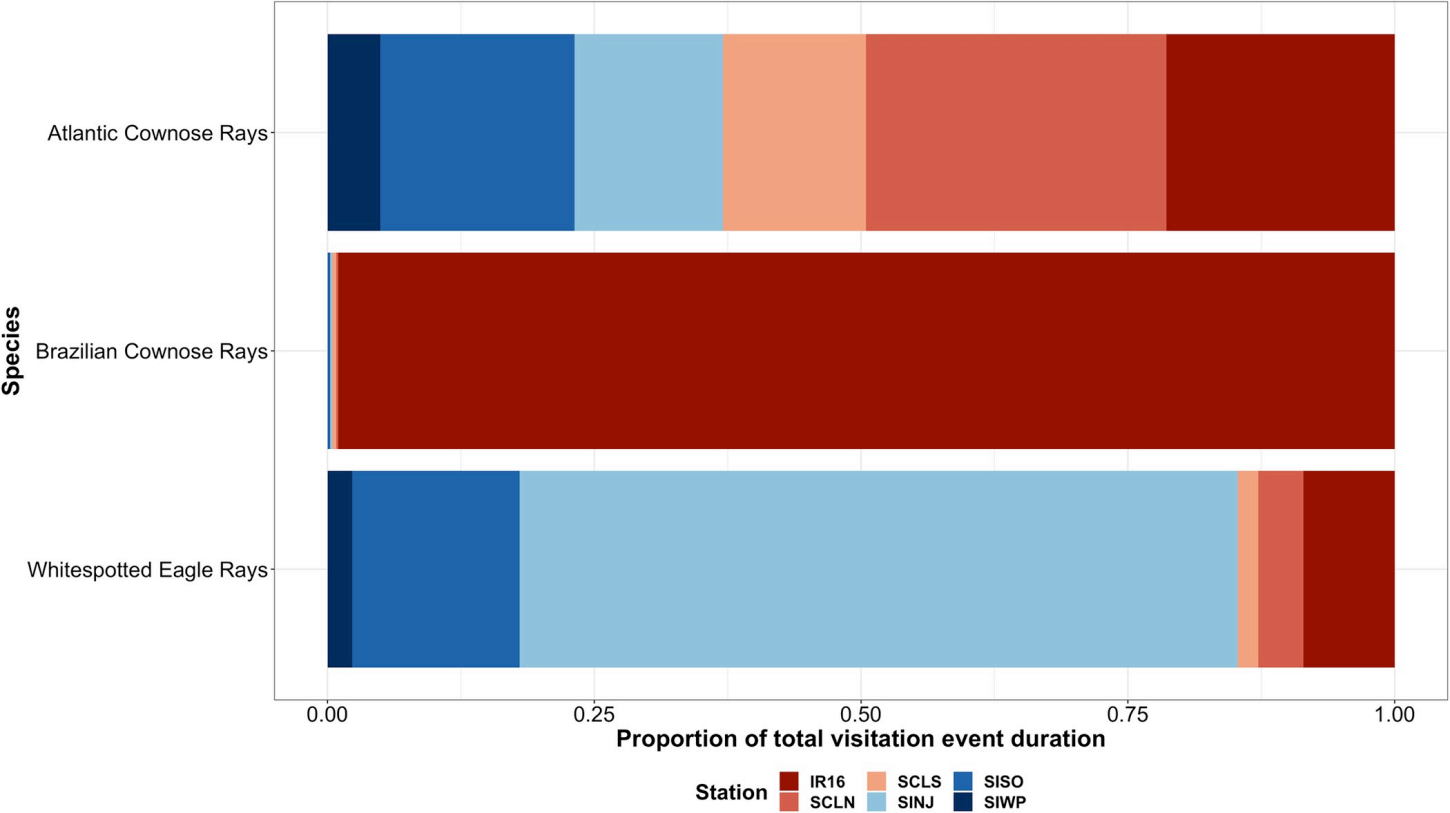
Proportion of visit duration at each receiver location for Brazilian cownose rays, Atlantic cownose rays, and whitespotted eagle rays.

There were 17,014 unique visits derived from 236,458 detections, and the number of visits ranged from 0–2,541 for the 38 tagged individuals considered (Table 1). Most of the visits occurred at SINJ with a total of 7,256 visits, but 97.3% of those were attributed to whitespotted eagle rays (Table 4). Similarly, the second highest number of visits occurred at SISO (n = 4,233), with 89.8% of those from whitespotted eagle rays. Receiver IR16 had the third highest number of visits, but most were from Brazilian cownose rays (48.7%), whereas whitespotted eagle rays made up 42.0%. Brazilian cownose rays spent >99.0% of the total visits at the IR16 receiver, whereas clam lease use was limited to 0.6% of their detected time (Fig 5). Conversely, Atlantic cownose rays spent most of their total visit time at SCLN (28.2%), but the total time spent at the clam leases accounted for 41.6% of their detected time. Finally, whitespotted eagle rays spent 85.3% of their visit duration near the inlet receivers, compared to 12.9% at the clam lease locations.

**Table 4:** Visit durations by species at each receiver. Number of unique individuals that exhibited visits described in the “Unique Tags” column. Number of visits denoted by (N). Total time is the summation of all visits. Time is reported in minutes.

	Brazilian Cownose Rays					Atlantic Cownose Rays					Whitespotted Eagle Rays				
Receiver	Unique Tags	Range (min)	Mean (min)	N	Total Time (min)	Unique Tags	Range (min)	Mean (min)	N	Total Time (min)	Unique Tags	Range (min)	Mean (min)	N	Total Time (min)
**IR16**	2	0.6–1554.7	63.1 ± 4.8	1,200	75,665.0	10	0.7–194.9	31.5 ± 2.6	230	7,239.9	16	0.7–604.5	34.1 ± 1.3	1,036	35,339.9
**SCLN**	1	1.6–24.1	10.6 ± 2.1	13	138.1	10	0.8–387.5	33.0 ± 2.5	289	9,536.3	16	0.6–220.1	19.8 ± 0.7	886	17,541.9
**SCLS**	1	1.9–34.4	14.7 ± 2.2	20	293.8	9	0.9–138.0	24.2 ± 1.9	188	4,541.9	15	0.8–207.1	30.0 ± 2.0	264	7,917.7
**SINJ**	1	2.5–66.3	14.2 ± 6.7	9	127.7	10	0.8–213.0	25.4 ± 2.7	186	4,715.4	20	0.6–927.4	39.4 ± 0.7	7,061	278,371.9
**SISO**	1	1.1–29.5	7.6 ± 1.5	25	190.6	13	0.6–298.3	15.3 ± 1.0	403	6,169.9	18	0.6–514.4	17.0 ± 0.4	3,805	64,827.4
**SIWP**	2	2.1–4.8	3.8 ± 0.9	3	11.4	11	0.6–200.1	12.8 ± 2.1	131	1,672.4	18	0.6–82.3	7.6 ± 0.2	1,265	9,584.5
**Total**	3	0.6–1554.7	60.2 ± 4.5	1,270	76,426.6	14	0.6–387.5	23.7 ± 0.9	1,427	33,875.9	20	0.6–927.4	28.9 ± 0.4	14,317	413,583.3

**Fig 6:**
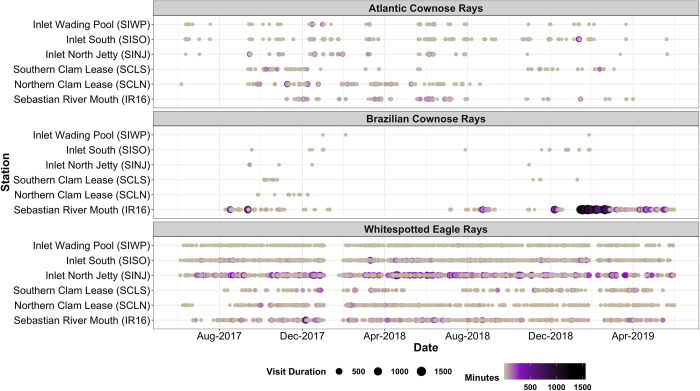
All visits by receiver location and species.

Whitespotted eagle rays exhibited the most visits, longest continuous visit duration, longest mean visit duration, and the greatest total time within range of the SINJ receiver (Table 4; Fig 6). Receiver IR16 had the second longest mean visit duration, and second longest continuous visit duration, but SISO had more visits and a greater total time spent. Receiver SCLS had the third longest mean visit duration but only had 264 visits and the least total time spent at 7,917.7 min. Atlantic cownose rays exhibited the most visits at the SISO receiver; however, the longest continuous visit duration, longest mean visit duration, and greatest total time was within range of the SCLN receiver (Table 4). Receiver IR16 had the second longest mean visit duration and the second greatest total time spent. Brazilian cownose rays exhibited the greatest continuous visit duration, longest mean visit duration, greatest number of visits and greatest total time at the IR16 receiver. The second longest mean visit duration and second greatest total time was observed at SCLS, but the second greatest continuous visit duration was observed at SINJ. Receiver SIWP displayed the shortest mean visit duration for all species considered. Visits were infrequently observed during summer months (June–August) during 2017 and 2018 for any species (Fig 6) at the clam leases.

Overall, 7,297 visits were above our long duration visit threshold of 17.1 min, potentially indicating interactions between the animals and the habitats in which the receivers were positioned. Brazilian cownose rays (n = 2) made 620 visits, Atlantic cownose rays (n = 12) made 567 visits, and collectively all 20 whitespotted eagle rays made 6,110 visits. Of 1,660 total visits at the two clam leases (SCLN and SCLS), 764 were above the long visit threshold. A Brazilian cownose ray (n = 1) made 12 visits, Atlantic cownose rays (n = 9) made 242 visits and whitespotted eagle rays (n = 16) made 510 visits.

### Environmental factors

**Fig 7:**
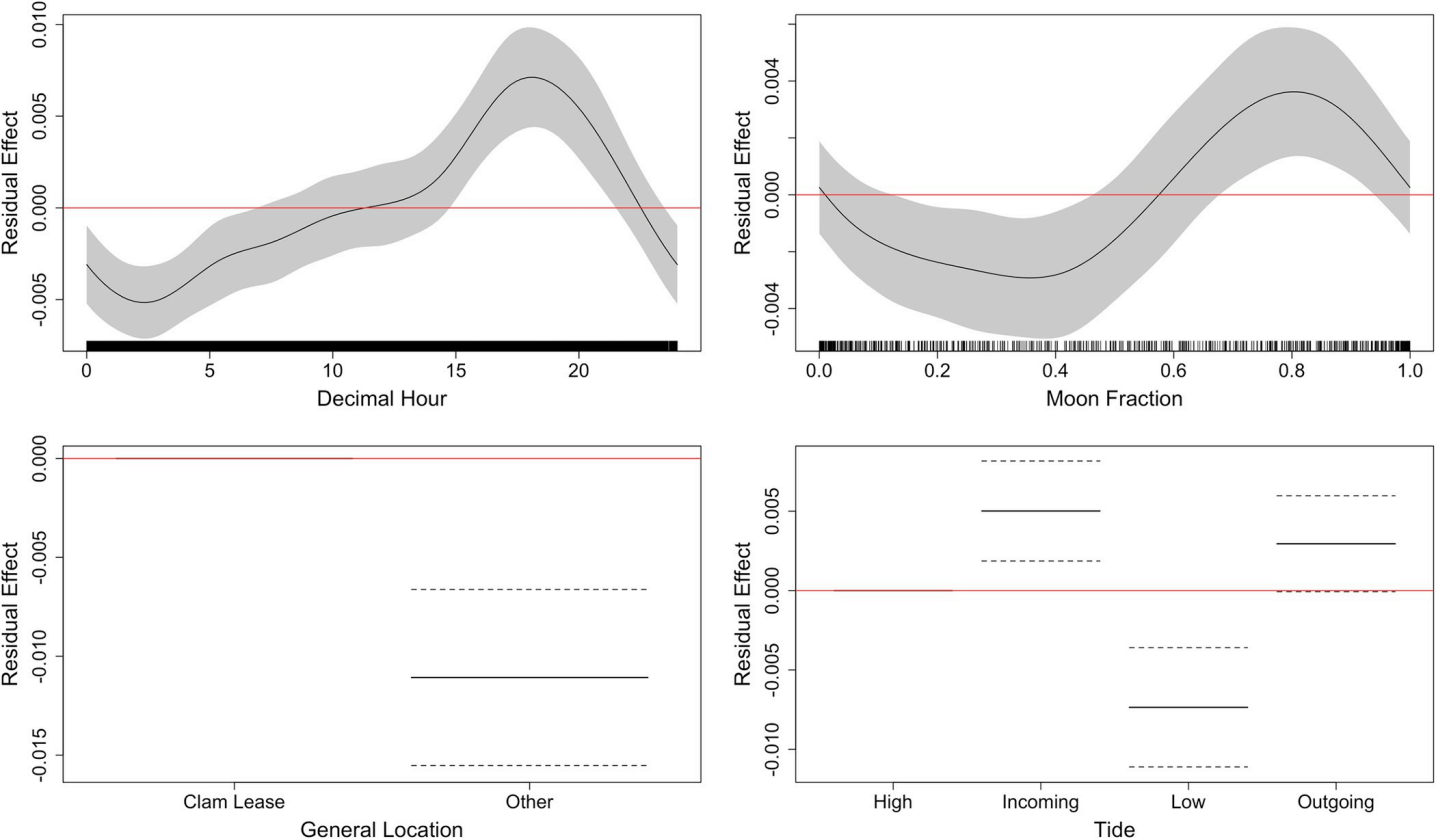
Generalized additive mixed effects model residual effect plots for the optimal model only including whitespotted eagle ray visit data. Effect size of smoothed variables in the optimal whitespotted eagle ray visit model. Response is visit duration in minutes. Effect size is noted by the black line, 95% SE by the gray band, positive/negative effect threshold by the red line at zero, and model input data values are black vertical hashes above the x-axis.

**Fig 8:**
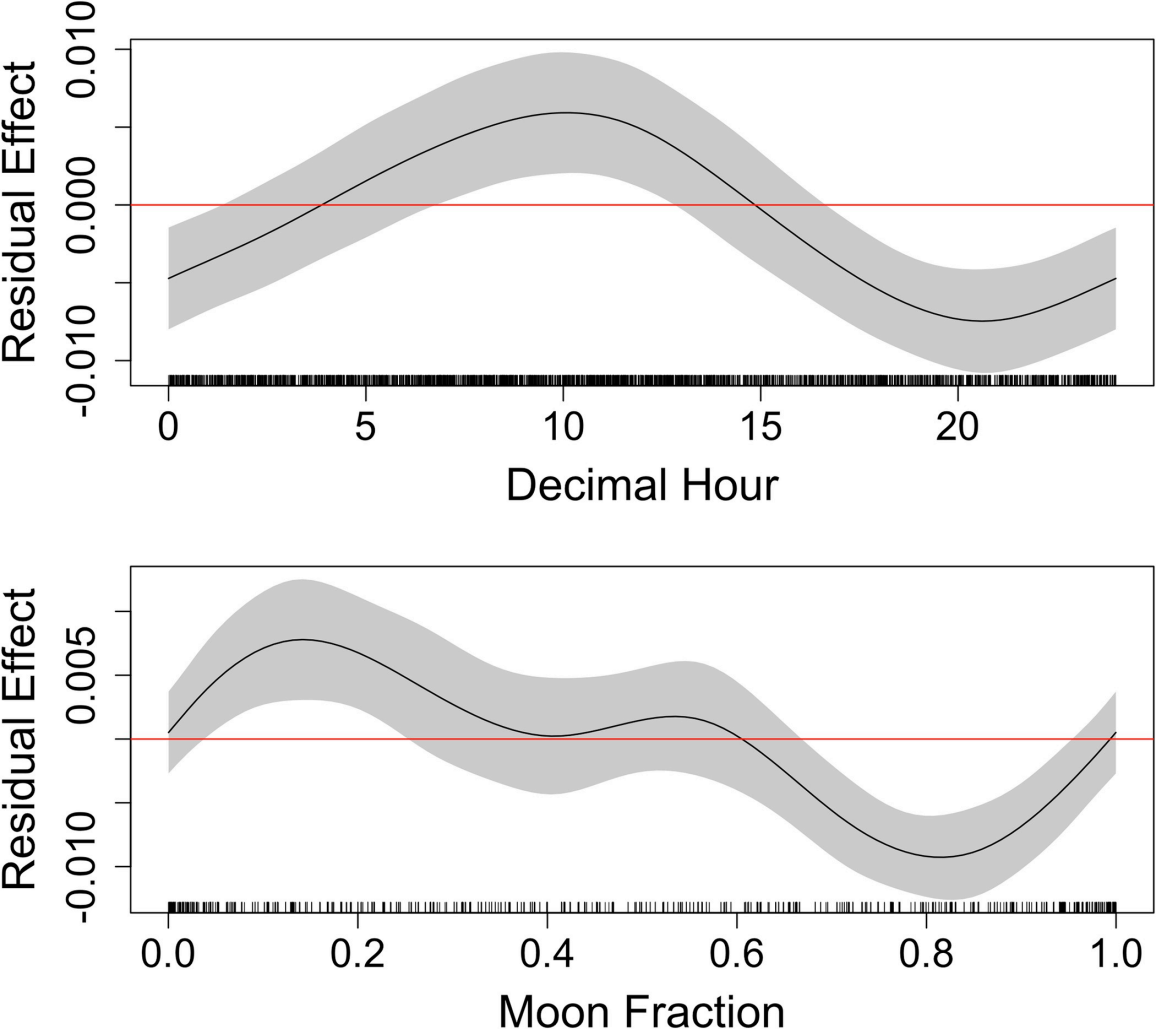
Generalized additive mixed effects model residual effect plots for the optimal model only including cownose ray visit data. Effect size of smoothed variables in the optimal cownose ray visit model. Response is visit duration in minutes. Effect size is noted by the black line, 95% SE by the gray band, positive/negative effect threshold by the red line at zero, and model input data values are black vertical hashes above the x-axis.

Collinearity was observed between temperature (°C) and oxygen saturation (%), but not dissolved oxygen (mg/L); thus, oxygen saturation was omitted from the model. The optimal GAMM for describing the effect of abiotic factors on visit duration of whitespotted eagle rays included the fixed effects: general location, tide, decimal hour and moon fraction (S2 Table, Table 5). Visit durations were significantly longer at clam leases, between 15:00 to 21:00 h, during waning phases of the moon (i.e., last quarter) and during flooding and ebbing tides (Fig 7). The optimal model for all cownose rays included decimal hour, and moon fraction (S3 Table, Table 6). Cownose rays exhibited significantly longer visits between 0700 to 1300 h, and during the waxing phases around the first quarter (Fig 8), though it is worthwhile to note that there was no significant difference in visit duration observed for general location (clam lease vs. other).

**Table 5:** Results of the optimal model for whitespotted eagle ray visit duration. The intercept represented accounts for both high tidal state and the general location of clam lease habitat; estimated degrees of freedom (edf), reference degrees of freedom (Ref.df).

**Covariate**		**edf**	**Ref.df**	**F**	**P**	R^2^ (adj.)
**Transmitter ID**		13.169	19	5.878	< 0.001	0.017
**Decimal Hour**		3.013	8	1.237	0.015	
**Moon Phase**		3.299	8	4.271	< 0.001	
	**Level**	**Coeff**	**SE**	**t value**		
**Intercept**	Intercept (High, Clam Lease)	0.042	0.003	13.663	< 0.001	
**Tide**	Incoming	0.004	0.002	2.028	0.043	
	Low	-0.007	0.002	-3.342	0.001	
	Outgoing	0.003	0.002	1.332	0.183	
**General Location**	Other	0.002	0.002	-5.612	< 0.001	

**Table 6:** Results of the optimal model for cownose ray visit duration; degrees of freedom (edf), reference degrees of freedom (Ref.df).

Covariate	edf	Ref.df	F	P	R^2^ (adj.)
**Transmitter ID**	7.775	16	8.554	< 0.001	0.063
**Decimal Hour**	4.073	8	8.189	0.001	
**Moon Phase**	2.431	8	1.207	0.007	

## Discussion

Overall, habitat use for whitespotted eagle rays and cownose rays suggested that neither species selected the clam lease habitats exclusively over other areas considered within the region, nor were the visit patterns substantially different from that observed at other receiver locations for cownose rays. Nevertheless, rays did occasionally spend extensive periods of time within range of the clam leases, often exhibiting longer visit durations than those observed at the inlet receivers SISO and SIWP. The longest continuous duration spent at the northern and southern clam leases were 387.5 and 207.1 min, respectively, demonstrating that individuals can remain within these areas for hours at a time. The long duration visits increase the likelihood in which the rays are interacting with, and potentially foraging upon, the clam leases. However, it is possible that the rays that visited clam leases may be feeding on other organisms that are attracted to the clams, as bivalve aquaculture farms have been known to change community structure and attract a wide variety of predatory gastropods, crabs, sea stars and other predatory invertebrates [4, 5], that may be an alternative attractant for cownose and whitespotted eagle rays given their diets [6–9, 11].

While most animals were collected near Sebastian Inlet, none were caught at or released within 2.8 km of the clam lease sites; yet, at least 73% of animals tagged were detected at least once on each clam lease receiver, and multiple individuals (n = 27, 96.4%) re-used these sites. In total, 26 individuals exhibited long duration visits (> 17.1 min) at the clam lease habitats. The repeated long duration visits indicate that these locations lie within movement corridors of both species within the IRL, although it is uncertain whether the clam leases are situated within their natural foraging habitats, which have yet to be described in this area. Use of the clam lease area was not consistent year-round, and varied seasonally, with substantially fewer detections and visits during the summer months. This suggests that clammers may be able to variably deploy anti-predator protections (e.g., polyester clam bags, cover netting, caging) [49] if ray predation is the primary concern.

### Species variation

Whitespotted eagle rays and cownose rays exhibited different distribution patterns within the Sebastian, FL region, with whitespotted eagle rays spending 85.6% of their detected time within range of the inlet receivers, whereas Brazilian cownose rays and Atlantic cownose rays spent 99.4% and 60.7% of their respective detected time within range of the lagoon receivers (Table 2). Of all tags detected, only one ray upon capture was visually determined to be pregnant (CNR 17). This individual was exclusively detected on IR16, with 35,585 detections amounting to 1,195 different visits. These detections occurred during fall of 2017, the late summer/early fall of 2018, and on 75% of days from January 2019 to June 2019. Within the last six months of the study, this individual exhibited a mean of 152.4 ± 10.6 min between subsequent visits, highlighting how active this animal was within the area. It is possible that IR16 is a potential area for cownose ray gestation and/or pupping. Similar spring parturition in Charlotte Harbor, FL has been described for *R*. *bonasus* [42] and captive *R*. *bonasus* also exhibited parturition between March–June [50]. Spring into summer also appears to coincide with pupping along the São Paula coast of southeastern Brazil, as high catch rates of juvenile cownose rays (both *R*. *bonasus* and *R*. *brasiliensis*) have occurred from early spring to fall, particularly from December to April [51]. Given differences in growth curves between *R*. *bonasus* and *R*. *brasiliensis* populations found in the western Atlantic Ocean [52] and Gulf of Mexico, Neer and Thompson [53] speculated that there may be a difference in parturition timing as well. Since CNR 17 was the only female *R*. *brasiliensis* caught that was visually described as pregnant, contrary to the 8 female *R*. *bonasus* caught that were not pregnant, it does appear that *Rhinoptera* spp. parturition timing may vary among species.

Presumably immature rays—disc width < 135 cm for whitespotted eagle rays, and < 70 cm for Atlantic cownose rays [9, 52, 54, 55]—did not use the Sebastian receiver array as extensively as larger conspecifics (Table 1, Fig 4). Unfortunately, due to small sample sizes and insufficient detections, we were unable to statistically assess variation in detection patterns across ontogeny. Juvenile whitespotted eagle rays (< 100 cm) were detected primarily on lagoon receivers, whereas adults (> 135 cm) on the inlet receivers, and subadults (100–135 cm) on a mix of both lagoon and inlet receivers. DeGroot et al. [28] found that juvenile whitespotted eagle rays used lagoon habitats more frequently than larger conspecifics, which is consistent with the information presented here. In our study, both juvenile cownose rays (< 50 cm) and subadults (50–70 cm) were each detected < 5 days with detections occurring in the inlets, while adults (> 70 cm) were primarily and regularly detected on the lagoon receivers. Given that mature cownose rays were detected primarily on lagoon receivers, and juveniles have few detections only occurring at the inlets, it is possible that juvenile cownose rays might be using areas further away from the inlet (i.e., northern or southern locations within the IRL) while evading the three lagoon receivers. Such behavior for juvenile cownose rays would not be unusual given their preference of lower salinities at early life stages [56], while evading the three lagoon receivers. This ontogenetic partitioning has also been proposed as a strategy to find predation refuge and to associate with finer sediment substrates where prey is likely more plentiful and accessible [56]. While we observed periodic presence of cownose rays via detections within the Sebastian acoustic receiver array, there were large undetected time periods that need to be further studied with an expanded receiver array within the IRL and offshore.

There was a large disparity in the number of detections and visits between whitespotted eagle and cownose rays. Despite similar sample sizes, cownose rays were detected less than whitespotted eagle rays and exhibited fewer visits. Cownose rays have been known to exhibit large-scale philopatric migrations, moving at the onset of summer from the IRL and Cape Canaveral, FL region up to Long Island, NY and New England [26, 57]. Conversely, Collins et al. [13] determined that cownose rays in southwest Florida might not exhibit the same long migrations observed in other regions. We observed periodic detections from cownose rays during the summer months in both 2017 and 2018, indicating that individuals do not always exhibit migratory behavior. Cownose rays were most present from September 2017 to January 2018, March 2018 to August 2018 and less commonly from September 2018 to March 2019 (Fig 4). One potential reason for the inconsistent cownose ray detections could be a result of mixed schools including both *R*. *bonasus* and *R*. *brasiliensis* in the Sebastian, FL region, though no *R*. *brasiliensis* were detected on the Sebastian receiver array from early February 2018 to June 2018. Weber et al. [58] documented a range expansion for *R*. *brasiliensis* to include parts of the western North Atlantic, up to Sebastian, FL. It is likely that these animals are staying within the IRL given the short durations between detections are not extensive enough to migrate to the northern extent of the Mid-Atlantic Bight, and thus would be using areas beyond the Sebastian receiver array. The most common detection period for a large majority of individuals was during the Fall of 2017, which is consistent with findings that cownose rays in southwest Florida were more frequently observed during the summer and fall months than during other seasons [59]. Within the study period, there were examples of synchronized movements in which cownose rays appeared to follow similar patterns when moving from September 2017 to January 2018, and again from March 2018 to June 2018. In contrast, a majority of whitespotted eagle rays were detected throughout the year, excluding those that only entered the system briefly. This is consistent with findings from DeGroot et al. [28], confirming that whitespotted eagle rays caught in the IRL are residential. While cownose and whitespotted eagle rays typically show disparate emigration and residency behaviors at a given time, we observed synchronous behavior across species in January 2018. We hypothesize that the synchronous behavior was in response to an abrupt and dramatic cold event, forcing the animals to seek thermal refuge in warmer offshore waters [60]. These differences in detections and migration behaviors between the two species imparts different likelihoods in their capacity to interact with shellfish enhancement activities.

### Environmental drivers

Generally, both species exhibited more detections during the night at the lagoon receivers and more during the day at the inlet receivers; however, cownose and whitespotted eagle rays exhibited longer visit patterns at different times within a 24 h period. These differences in visits are likely a result of differing movement strategies and/or habitat preferences between the two species. Whitespotted eagle rays are observed in shallower waters at night and deeper waters during the day [11, 14, 61], which has been hypothesized to be a thermoregulatory behavior [11]. Behavioral thermoregulation, or behavioral strategies used to maintain body temperature outside of thermogenesis, has been observed in other batoid rays such as bat rays (*Myliobatis californica*) [62] in Tomales Bay, California. Bat rays exhibited patterns opposite to that of whitespotted eagle rays, in which they were observed in shallow, warmer waters from early morning to midday [62]. These diel patterns were suggested as a foraging strategy in which the rays moved to shallow, warm waters to feed on diurnally active burrowing mollusks, and then retreat to cooler waters to rest and digest [62]. While the diel patterns observed between bat rays and whitespotted eagle rays are different, this may be less of a difference in circadian rhythm and more a difference in the bathymetry causing different temperature regimes, and thus shifting the ideal times for foraging behaviors. Similarly, whitespotted eagle ray presence in shallow water has been speculated to be a foraging strategy to encounter mollusks in shallow, warm waters. Given that clam leases are normally 1–2 m in depth [38] and that there is a higher proportion of detections at the lagoon locations and longer visits at night, it is possible that clammers (who primarily work during day) are underestimating how often rays are interacting with areas used for shellfish aquaculture. However, it is worthwhile to consider that the proportion of whitespotted eagle ray visits at the clam leases only accounts for 6.2% of their total time. Alternatively, the diel behaviors observed for whitespotted eagle rays (i.e., retreating to deeper waters) could also represent a negative response to boat traffic in the area as demonstrated by spotted eagle rays (*A*. *ocellatus*) in French Polynesia [23].

Previous research suggests that cownose rays are most active periodically throughout the day and for a few hours at night [59] while other studies suggest no diel patterns [13, 57]; however, these apparent differences may be a result of using different tagging methodology, different study habitats, and potentially disparate populations with different behaviors. Our results suggested a slight association for more extended visits around late morning, which could align with foraging behaviors. Ehemann et al. [63] assessed the diet of the golden cownose ray (*Rhinoptera steindachneri*) and in response to observing a high overall vacuity index for animals caught predominantly at night, hypothesized that *R*. *steindachneri* primarily feed during daytime, around 12:00 h. More clarity on the diel feeding behaviors of *Aetobatus* sp. and *Rhinoptera* spp. can be gained by continuing tagging and monitoring efforts in the Sebastian, FL area. Furthermore, conducting body kinematic studies to understand feeding patterns of both genera could provide insight on the stability of these feeding behaviors to acute and chronic perturbations (e.g., boat traffic).

Given the disparate temporal associations in visit duration between cownose and whitespotted eagle rays, we posit that the two species are using the habitats differently due to limitations imposed by abiotic factors as well. Moon phase was a significant component to the models for both species; however, while both exhibited longer visits during neap tides, cownose rays had stronger associations with waxing phases near the first quarter, and whitespotted eagle rays with waning phases around the third quarter. Catch rates of whitespotted eagle rays across lunar phases has been greatest during waning lunar phases, followed by full moon [64]. Whitespotted eagle rays may be spending more time in these areas when there is still available lunar light, though limiting their exposure to visual predators that forage in shallower waters aided by the full moon. Tidal effects on elasmobranch presence varies by species, as evidenced by variability in modeled catch data [65]. Here, the rays exhibited contrasting activity patterns during neap tides, whereby longer visits occurred during waxing and waning phases for cownose and whitespotted eagle rays, respectively. Similarly, fisherman have described anecdotal information suggesting whitespotted eagle ray catches were negatively related to cownose ray presence [64]. These contrasting visit patterns may be a result of interspecific competition due to their shared semipelagic nature and likely comparable trophic levels confirmed via overlapping isotopic niches observed in Australian cownose rays (*Rhinoptera neglecta*) and spotted eagle rays (*A*. *ocellatus*) [66] and similar diet compositions [6–9, 11].

In the IRL, the mechanism for neap tide-driven visits may be less driven by light availability, but rather with a slightly lower tidal range reducing the velocity of water movement. The IRL has been described as microtidal, displaying overall intertidal amplitudes of 5–10 cm in the central portion influenced by the Sebastian Inlet [67], emphasizing that there is little difference between spring and neap tidal water volume within the lagoon [68]. However, tide-driven current speeds vary greatly between spring and neap tides, with spring tide and neap tide currents two km north of the Sebastian inlet flowing up to **±** 18 cm s^-1^ and **±** 10 cm s^-1^, respectively [69]. The decreased current speed observed during neap tides may allow rays to maintain their lateral position more consistently, and thus facilitate longer visits.

Tide was a significant factor for whitespotted eagle rays with longer visits during flooding and ebbing tides, but not during low tide conditions. However, despite being a non-significant component for the cownose ray model, cownose rays exhibited longer visits during low tide, followed by flooding and ebbing tidal cycles. Cownose rays may be moving throughout the lagoon in a positive relationship to the tide, like that of other elasmobranch species including juvenile freshwater sawfish (*Pristis microdon*) [70] and blacktip sharks (*C*. *limbatus*) [71]. This could potentially be a feeding strategy whereby cownose rays are able to forage in the lagoon and up the Sebastian River to access shallow-water prey items [72] that would otherwise be inaccessible during low tide. Alternatively, these movements may be a strategy to avoid predators. Using baited remote underwater videos, southern stingrays present in marine reserves were observed more frequently in shallow flats and avoided deeper areas that had a greater predator presence; however, southern stingrays in fished locations were observed in the deeper, forereef habitats, suggesting that predator-presence influenced movements and habitat use [73]. This predation-avoidance behavior may provide reasoning for why cownose rays were detected far less than whitespotted eagle rays in our study region yet exhibited visits at lagoon receivers primarily during low tide. Long duration visits displayed by whitespotted eagle rays during flooding and ebbing tides may be a result of specific foraging behaviors during tidal cycles or it could be an energetic strategy. Whitespotted eagle rays exhibited tidal-driven behavioral patterns, primarily foraging during high falling tide with little horizontal movement to other regions and exhibited a “resting phase” during low rising tide, where the rays faced into the current [74]. Similarly, DeGroot et al. [14] found that whitespotted eagle rays were primarily in channels during ebbing and low tides, with channels having the lowest rate of movement for all locations considered. Alternatively, this may be an energetics strategy like rheotaxis observed in grey reef sharks (*Carcharhinus amblyrhynchos*) during ebbing and flooding tides, utilizing updraft zones and shuttling within a school [75]. Since 85% of whitespotted eagle ray detected time was observed within the inlets, and their longer visits primarily occurred during ebbing and flooding tides, it is likely that these animals are relying on the inlet habitats for similar foraging or energetic strategies.

### Caveats and future directions

This study not only emphasized the use of rays around clam lease habitats, but it also provided insight into the difference in habitat use of inlet, river mouth and clam lease habitats for both cownose and whitespotted eagle rays. To accomplish this, we opportunistically integrated network receivers that were already deployed by other working groups within the area to act as reference receivers. While these receivers provided sufficient regional coverage and functionally contrasting habitats, future studies should consider reference locations with environments more comparable to the clam lease sites, such as similar benthic habitat, distance to shore and distance to inlet. Environmental conditions, such as water depth and current speeds can impact detection ranges, with higher proportions of detections in unstratified, deeper waters [76, 77] and lower current speeds having been observed [77]. Both factors could have biased detections for the deeper, inlet locations, or during periods of low current flow near the lagoon sites, or during tidal changes. Similarly, areas of high boat noise (e.g., boat channels, or highly frequented areas like clam leases) can limit detection capability [78]. Alternatively, another method that is commonly used to assess fine-scale movements is a gridded receiver array [78], which helps triangulate the location of a tagged animal within the study location. This technique has been used to quantify foraging behaviors and determine potential prey items of the Atlantic sturgeon (*Acipenser oxyrinchus*) by pairing activity to benthic habitat within the array [79], and has been done similarly with whitespotted eagle rays in Bermuda [11]. By employing a positioning array within the clam lease habitats, movements of invertivores and potential prey could be tracked and quantified, revealing how they are using the area at a fine scale. Ideally, such work would be conducted in a before-after-control-impact (BACI) study design to fully assess the bi-directional relationship of clam leases and community assemblages, though established clam leases can and should still have their ecological role examined.

We acquired environmental data from a central location in the IRL in Sebastian, FL; however, these data may not accurately reflect environmental conditions for all receiver locations, including the clam leases. Future studies should consider the use of deployable environmental monitoring tools or deploy receivers with temperature logging capabilities to collect more fine-scale environmental data. Additionally, the tidal offsets used were calculated based on a consistent rate of movement and the shortest distance between receivers but did not account for how water rate of movement will vary based on the region’s bathymetry, or how the rate varies throughout the tidal cycle.

The generalized additive mixed models for this study provided valuable insight into ray visit patterns during a wide variety of abiotic conditions; however, the low R^2^ values suggest that the variables examined do not comprehensively explain the variation in habitat use. One potentially influential unaccounted factor could be the deployment of the clam stocks themselves; however, that information was not available. Since all leases are individually owned, lease owners can deploy and retrieve clams at leisure. The two clam lease receivers (SCLN and SCLS) were deployed in partnership with Orchid Island Shellfish Company, Inc., as the leases that the company uses are consistently active. Various factors such as differing clam culture strategies, frequency of deployment and retrieval, as well as abundance of clams present at other leases may influence visit behavior. Regardless, the diel hydrological patterns between the inlet and lagoon are significant drivers of ray movements to the areas and thereby influence both species’ potential to interact with the clam leases.

Overall, we found that cownose and whitespotted eagle rays, two species of rays with the potential to interact with shellfish enhancement activities, are using IRL clam lease habitats differently and the habitats used are dependent on time of day. Despite less frequent use when compared to other reference receiver locations, both cownose and whitespotted eagle rays exhibited longer visits at clam lease sites than some locations considered and were observed within the clam lease regions for extended periods of time. These interactions confirm clammer observations within the area, and thus highlight the need to understand if rays are actively interacting with the clam leases, or if they may be attracted to other organisms nearby.

## Supporting information

S1 Table Summary information from the generalized linear mixed effects model (GLMM) assessing detection counts by the interaction term of time of day, station and sex for Atlantic cownose and whitespotted eagle rays. (DOCX)

S2 Table Model selection outputs for a generalized additive mixed effect model for whitespotted eagle rays. Transmitter ID was included in the top five models as a significant effect. The ideal model fit for the data is denoted by (*) and was selected by Akaike’s information criterion (AICc).
(DOCX)

S3 Table Model selection outputs for a generalized additive mixed effects model for cownose rays (*Rhinoptera* spp.). Transmitter ID was included in the top five models as a significant effect. The ideal model fit for the data is denoted by (*); Akaike’s information criterion (AICc).
(DOCX)
