# Supplementary material for: Visitation patterns of two ray mesopredators at shellfish aquaculture leases in the Indian River Lagoon, Florida
Source: PLoS One. 2023 May 4;18(5):e0285390. doi: 10.1371/journal.pone.0285390 (PMC10159191; doi:10.1371/journal.pone.0285390)
Supplement: S1 Table — (DOCX) [file pone.0285390.s001.docx]

**Table S1: Summary information from the generalized linear mixed effects model (GLMM) assessing detection counts by the interaction term of time of day, station and sex for Atlantic cownose and whitespotted eagle rays.**

|  | Atlantic Cownose Rays | | | | Whitespotted Eagle Rays | | | |
| --- | --- | --- | --- | --- | --- | --- | --- | --- |
| Random Effects | Variance | Std. Dev. |  |  | Variance | Std. Dev. |  |  |
| Transmitter ID | 0.109 | 0.330 |  |  | 0.229 | 0.344 |  |  |
| Coefficients | **Estimate** | **SE** | **Z value** | **P** | **Estimate** | **SE** | **Z value** | **P** |
| Intercept (Day, IR16, Female) | 1.613 | 0.130 | 12.380 | < 0.001 | 2.206 | 0.124 | 17.825 | < 0.001 |
| Night, IR16, Female | 0.325 | 0.047 | 6.941 | < 0.001 | 0.227 | 0.019 | 11.696 | < 0.001 |
| Day, SCLN, Female | 0.039 | 0.047 | 0.822 | 0.411 | -0.484 | 0.030 | -15.977 | < 0.001 |
| Day, SCLS, Female | -0.054 | 0.060 | -0.893 | 0.372 | -0.287 | 0.069 | -4.182 | < 0.001 |
| Day, SINJ, Female | -0.245 | 0.053 | -4.620 | < 0.001 | 0.277 | 0.017 | 16.197 | < 0.001 |
| Day, SISO, Female | -0.230 | 0.046 | -5.055 | < 0.001 | -0.223 | 0.020 | -11.397 | < 0.001 |
| Day, SIWP, Female | -0.324 | 0.056 | -5.826 | < 0.001 | -0.984 | 0.030 | -33.280 | < 0.001 |
| Day, IR16, Male | -0.061 | 0.207 | -0.294 | 0.769 | -0.399 | 0.160 | -2.492 | 0.013 |
| Night, SCLN, Female | -0.196 | 0.062 | -3.147 | 0.002 | -0.148 | 0.041 | -3.632 | < 0.001 |
| Night, SCLS, Female | -0.185 | 0.079 | -2.358 | 0.018 | 0.060 | 0.088 | 0.687 | 0.492 |
| Night, SINJ, Female | 0.252 | 0.072 | 3.497 | < 0.001 | -0.220 | 0.022 | -10.126 | < 0.001 |
| Night, SISO, Female | -0.214 | 0.070 | -3.055 | 0.002 | -0.241 | 0.026 | -9.213 | < 0.001 |
| Night, SIWP, Female | -0.169 | 0.100 | -1.694 | 0.090 | -0.217 | 0.046 | -4.695 | < 0.001 |
| Night, IR16, Male | -0.420 | 0.091 | -4.620 | < 0.001 | 0.027 | 0.041 | 0.646 | 0.518 |
| Day, SCLN, Male | -0.094 | 0.090 | -1.045 | 0.296 | 0.453 | 0.048 | 9.414 | < 0.001 |
| Day, SCLS, Male | -0.281 | 0.113 | -2.492 | 0.013 | 0.451 | 0.083 | 5.458 | < 0.001 |
| Day, SINJ, Male | -0.132 | 0.101 | -1.315 | 0.188 | 0.357 | 0.036 | 9.904 | < 0.001 |
| Day, SISO, Male | -0.139 | 0.093 | -1.497 | 0.135 | 0.212 | 0.038 | 5.576 | < 0.001 |
| Day, SIWP, Male | -0.246 | 0.133 | -1.852 | 0.064 | 0.335 | 0.048 | 7.032 | < 0.001 |
| Night, SCLN, Male | 0.506 | 0.116 | 4.376 | < 0.001 | 0.092 | 0.061 | 1.505 | 0.132 |
| Night, SCLS, Male | 0.842 | 0.139 | 6.058 | < 0.001 | -0.352 | 0.105 | -3.366 | < 0.001 |
| Night, SINJ, Male | -0.178 | 0.162 | -1.096 | 0.273 | -0.097 | 0.043 | -2.268 | 0.023 |
| Night, SISO, Male | 0.529 | 0.129 | 4.082 | < 0.001 | -0.248 | 0.047 | -5.260 | < 0.001 |
| Night, SIWP, Male | 0.193 | 0.203 | 0.949 | 0.343 | -0.196 | 0.067 | -2.941 | 0.003 |
