# Supplementary material for: Visitation patterns of two ray mesopredators at shellfish aquaculture leases in the Indian River Lagoon, Florida
Source: PLoS One. 2023 May 4;18(5):e0285390. doi: 10.1371/journal.pone.0285390 (PMC10159191; doi:10.1371/journal.pone.0285390)
Supplement: S2 Table — Transmitter ID was included in the top five models as a significant effect. The ideal model fit for the data is denoted by (*) and was selected by Akaike’s information criterion (AICc). (DOCX) [file pone.0285390.s002.docx]

**Table S2: Model selection outputs for a generalized additive mixed effect model for whitespotted eagle rays.** Transmitter ID was included in the top five models as a significant effect. The ideal model fit for the data is denoted by (*) and was selected by Akaike’s information criterion (AICc).

| Model log (Gamma) | Intercept | df | LogLik | AICc | ∆AICc | Weight |
| --- | --- | --- | --- | --- | --- | --- |
| *f(Transmitter ID) + f(General Location) + f(Tide) + s(Decimal Hour) + s(Moon Phase) | 0.042 | 9 | 20864.49 | -41711.0 | 0.00 | 0.99 |
| f(Transmitter ID) + f(General Location) + f(Tide) + s(Moon Phase) | 0.042 | 8 | 20850.17 | -41684.3 | 26.63 | 1.65 e^-6^ |
| f(Transmitter ID) + f(General Location) + f(Tide) + s(Decimal Hour) | 0.042 | 8 | 20793.93 | -41571.9 | 139.11 | 6.21 e^-31^ |
| f(Transmitter ID) + f(General Location) + s(Decimal Hour) + s(Moon Phase) | 0.044 | 6 | 20774.73 | -41537.5 | 173.50 | 2.11 e^-38^ |
| f(Transmitter ID) + f(General Location) + f(Tide) | 0.042 | 7 | 20773.47 | -41532.9 | 178.03 | 2.19 e^-39^ |
