# Supplementary material for: Visitation patterns of two ray mesopredators at shellfish aquaculture leases in the Indian River Lagoon, Florida
Source: PLoS One. 2023 May 4;18(5):e0285390. doi: 10.1371/journal.pone.0285390 (PMC10159191; doi:10.1371/journal.pone.0285390)
Supplement: S3 Table — Transmitter ID was included in the top five models as a significant effect. The ideal model fit for the data is denoted by (*); Akaike’s information criterion (AICc). (DOCX) [file pone.0285390.s003.docx]

**Table S3: Model selection outputs for a generalized additive mixed effects model for cownose rays (Rhinoptera spp.).** Transmitter ID was included in the top five models as a significant effect. The ideal model fit for the data is denoted by (*); Akaike’s information criterion (AICc).

| Model log (Gamma) | Intercept | df | LogLik | AICc | ∆AICc | Weight |
| --- | --- | --- | --- | --- | --- | --- |
| *f(Transmitter ID) + s(Decimal Hour) + s (Moon Phase) | 0.045 | 5 | 4017.545 | -8025.067 | 0.000 | 0.99 |
| f(Transmitter ID) + f(Tide) + s(Decimal Hour) + s (Moon Phase) | 0.043 | 8 | 4013.005 | -8009.957 | 15.110 | 5.23 e^-3^ |
| f(Transmitter ID) + f(General Location) + s(Decimal Hour) + s (Moon Phase) | 0.038 | 6 | 4002.215 | -7992.399 | 32.668 | 8.05 e^-7^ |
| f(Transmitter ID) + f(General Location) + f(Tide) + s(Decimal Hour) + s (Moon Phase) | 0.036 | 9 | 3999.810 | -7981.553 | 43.514 | 3.56 e^-9^ |
| f(Transmitter ID) + s (Moon Phase) | 0.043 | 4 | 3992.315 | -7976.616 | 48.451 | 3.01 e^-10^ |
